# Gestational Weight Gain Charts by Gestational Age and Body Mass Index for Chinese Women: A Population-Based Follow-up Study

**DOI:** 10.2188/jea.JE20180238

**Published:** 2020-08-05

**Authors:** Aiqun Huang, Yanhui Xiao, Huanqing Hu, Wei Zhao, Qi Yang, Weixiao Ma, Linlin Wang

**Affiliations:** 1 National Center for Women and Children’s Health, Chinese Center for Disease Control and Prevention, Beijing, China; 2 Institute of Reproductive and Child Health, National Health Commission Key Laboratory of Reproductive Health, Department of Epidemiology and Biostatistics, School of Public Health, Peking University Health Science Center, Beijing, China

**Keywords:** gestational weight gain, Chinese BMI standard, Z-score charts, weight management, internal and external validation

## Abstract

**Background:**

Establishment of an unbiased association between gestational weight gain (GWG) and perinatal health is urgently needed in China, which has the largest population in the world. Our study aimed to create weight-gain-for-gestational-age charts using early pregnancy body mass index (BMI) to present selected percentiles of GWG in China.

**Methods:**

A population-based follow-up study was conducted based on the Maternal and Newborn Health Monitoring System, which recruited 132,835 pregnant women between October 2013 and September 2016 in 12 districts/counties of 6 provinces in China. Multilevel analyses and restricted cubic splines were performed to model the longitudinal repeated maternal weight gain measurements and obtain smoothed curves for GWG. The internal and external validation of each model was also assessed.

**Results:**

To develop models of GWG, 34,288 women were included. Smoothed percentiles of GWG in the 3rd, 10th, 50th, 90th, and 97th percentiles were estimated for each week of gestation. The median figures for GWG were 15.0 kg, 14.4 kg, 13.5 kg, and 12.1 kg in underweight, normal weight, overweight, and obese women, respectively, at 40 weeks. Of all the weight measurements, more than 70% and 95% fell within the expected 1 to 2 standard deviations, respectively. To accomplish external validation of the models, 20,458 women were included. The specificities of measurements in the 5th, 10th, 15th, 25th, 75th, 85th, 90th, and 95th percentiles in four BMI categories were between 90% and 100%.

**Conclusions:**

The population-based gestational weight gain Z-score charts performed well in providing guidance regarding expected gestational weight gain in Chinese women.

## INTRODUCTION

Gestational weight gain (GWG) that is either insufficient or excessive has been associated with increased risks of short-term adverse outcomes (such as preterm birth, neonates who are small/large for gestational age, low birth weight/macrosomia, and cesarean delivery), as well as long-term adverse outcomes (such as maternal postpartum weight retention, childhood obesity, and adolescent cardiometabolic risk).^1^^–^^8^ Weight management by women during pregnancy is of great benefit to prevent adverse pregnancy outcomes induced via insufficient or excessive GWG.

A guideline for appropriate GWG is necessary to provide a reference for weight management in pregnant women. The Institute of Medicine (IOM) proposed guidelines for the rate of GWG and total GWG by a woman’s pre-pregnancy body mass index (BMI) in 2009.^9^ However, the IOM’s guidelines may have some limitations in clinical application.^10^ Because total GWG is strongly correlated with gestational duration and rates of GWG vary with gestational age (GA), usage of the IOM guidelines may introduce bias into weight management of pregnant women.^11^ Therefore, a new measure for monitoring pregnancy weight gain by GA is needed to control GWG more accurately.

Z-score charts of average maternal weight gain by GA can be created just as the growth standards for children aged 0 to 5 years were created, which have now been applied in many countries worldwide.^12^ Z-score charts can provide mean values and standard deviations (SDs) for weight gain throughout gestation, specific to gestational week, which would be used to calculate Z-scores and percentiles for GWG by GA. New measures have been developed in the United Kingdom,^13^ the United States,^12^^,^^14^ Sweden,^15^ and Malawi.^16^ However, most of these were established with small sample sizes and were not population based, not stratified by pre-pregnancy BMI values, and/or not verified for validity. The only standard that was based on population and stratified by pre-pregnancy BMI values was created in Sweden; however, external validation was not provided,^15^ which is necessary for clinical application. In addition, all of these Z-score charts are based on the World Health Organization (WHO) BMI categories, which are not available specifically for Asians, who have a higher body fat ratio and risk of obesity-related diseases than Europeans with the same BMI.^17^^,^^18^ Thus, there are different cut-offs for BMI categories for people of Asian and European descent.

Because China has the largest population in the world, there is an urgent need to establish Z-scores for GWG in Chinese women by GA. Therefore, we performed this study to create Z-score charts based on Chinese BMI categories in a population-based follow-up study of Chinese women. In addition, we assessed the internal and external validation of the new charts. We investigated healthy pregnant women with good pregnancy outcomes, according to recommendations by the WHO.^19^

## METHODS

### Study population

Data on the study population were obtained from the Maternal and Newborn Health Monitoring System (MNHMS) at the National Center for Women and Children’s Health, Chinese Centers for Disease Control and Prevention. The MNHMS was established to comprehensively monitor the prenatal health care and pregnancy outcomes information of pregnancy women. Participants resided in 12 districts/counties of 6 provinces: Liaoning, Hebei, Fujian, Guangdong, Sichuan, and Yunnan. A total of 132,835 pregnant women had been recruited between October 2013 and September 2016. One city from each province was selected randomly, and then two districts/counties from each city were selected randomly via three-stage cluster sampling. All pregnant women in 12 districts/counties were included if they had resided for at least 6 months at one of the 12 study sites and had been recruited at their first prenatal care visit. The data of MNHMS contained all maternal information obtained prior to pregnancy, at all prenatal care visits, at delivery, and during neonatal care (specific data collected are detailed in eAppendix 1). This study was exempt from both informed consent and ethics committee approval by the Ethics Committee for Human Subjects Studies of the National Center for Women and Children’s Health, Chinese Center for Disease Control and Prevention.

The data from the study population were divided into two sets. A training sample set was collected from women who delivered between October 2013 and September 2015, and a validation sample set was collected from women who delivered between October 2015 and September 2016. The training sample set was used to develop GWG models, and the validation sample set was used to test the validity of the GWG models.

Both data sets were created according to the same inclusion and exclusion criteria. Women were included if their neonates were singleton, non-anomalous, full-term, live births. Women were excluded if prenatal weights had been measured fewer than two times or if the first weight measurement had not been taken during early pregnancy (<13 completed weeks). Women were excluded if any weight observations that were ⩾4 SDs from the expected weight on the basis of the woman’s weight at the previous visit. In addition, women with pre-existing hypertension or type 1 or 2 diabetes mellitus, as well as women with no data on birthweight or with infants who were small for GA (<the 10th percentile of the average birthweight of the study population) or large for GA (>the 90th percentile of the average birthweight of the study population), were excluded from the analyses.

### Measurements

Maternal height (in centimeters) and weight (in kilograms) were measured at the first prenatal care visit (<13 completed weeks) to calculate an early pregnancy BMI (kg/m^2^). The Chinese BMI category standards of the Working Group on Obesity in China^20^ were used to categorize women as underweight (<18.50 kg/m^2^), normal weight (18.50–23.99 kg/m^2^), overweight (24.00–27.99 kg/m^2^), or obese (⩾28.00 kg/m^2^). Maternal weight should be assessed at every routine prenatal visit. Gestational weight gain (in kilograms) was calculated as the difference between the measured weight at the time of a prenatal care visit or at delivery and the measured weight at the first prenatal visit. Gestational age was estimated based on the last menstrual period and ultrasound.

### Statistical analyses

We created four separate models for each BMI category (underweight, normal weight, overweight, and obese) and applied multilevel (random-effects) analysis to model the longitudinal repeated maternal weight gain measurements as a function of GA. Weight gain measurements were log transformed (natural log) before modeling to ensure homoscedasticity of any residual errors and normality of distribution of the data. To prevent negative values on the charts, a constant was added to all weight measurements to shift the minimum value of the distribution to 1 before we modeled the data on the log scale. The models were specified with two levels (measurements within and between women) and random intercepts and random slopes, which provided more flexible estimates of weight gain patterns. To obtain an equation for week-specific SDs, an unstructured covariance matrix was ensured from the multilevel model. We modeled GA using restricted cubic splines to obtain smoothed curves for GWG.^11^ The number and location of the spline knots were chosen according to the Akaike information criterion (AIC), and a minimum AIC value for the model meant that the goodness of fit is optimal.

A sensitivity analysis was performed to explore the possibility of misclassification bias that might have occurred through for the use of early pregnancy BMI rather than pre-pregnancy BMI. We performed a post hoc analysis to estimate the proportion of women that might have been misclassified because of a weight gain of 2 kg during the first trimester^8^^,^^12^ (for example, women who would have been in the underweight group but were actually in the normal weight group because of weight gain during the first trimester). After regrouping, the data were modeled with the same strategy, and the new Z-score charts were compared with those obtained from original classification.

We assessed the internal validation of the model with the training sample set by visually comparing the fit of the predicted means and SDs to the crude data and calculating the percentages of crude weight gain measurements that fell within the predicted limits for 1 and 2 SDs (where 68% and 95%, respectively, would be expected in a perfect model). Moreover, we assessed the external validation of the model using the validation sample set that was collected during the third study year. We assessed the external validation of the model with two methods. First, we use the same method as internal verification to compare the fit of the models that developed by training set to the crude weight gain data of validation set. Then to further explore the external validation, the sensitivity and specificity of each BMI category model were calculated to identify selected percentiles for GWG and the Youden index (Youden index = sensitivity + specificity − 1) was also calculated. Because no optimal cut-offs of GWG Z-score charts were available, we selected 5th, 10th, 15th, 25th, 75th, 85th, 90th, and 95th percentiles to assess. In order to obtain the sensitivity and specificity, the Z-scores of the validation samples were calculated with the developed GWG models (a woman’s observation value of GWG was added to the constant and the sum was transformed into a log scale; then, according to the GA and early pregnancy BMI of the pregnant woman, the estimated mean and SD were given to calculate Z-scores). The “gold standard positive” was defined as the calculated Z-score < the expect x-th percentile (estimated by the model), and “test positive” was defined as the calculated Z-score < observed x-th percentile (of Z-score of the validation set). Taking the 10th percentile as an example, “gold standard positive” was that if the calculated Z-score was less than the expected 10th percentile, and “test positive” was that if the calculated Z-score was less than observed 10th percentile. All analyses were performed using STATA Software, version 13.0 (StataCorp LP, College Station, TX, USA).

## RESULTS

### Developing models of weight gain for gestational age

**Figure 1.**
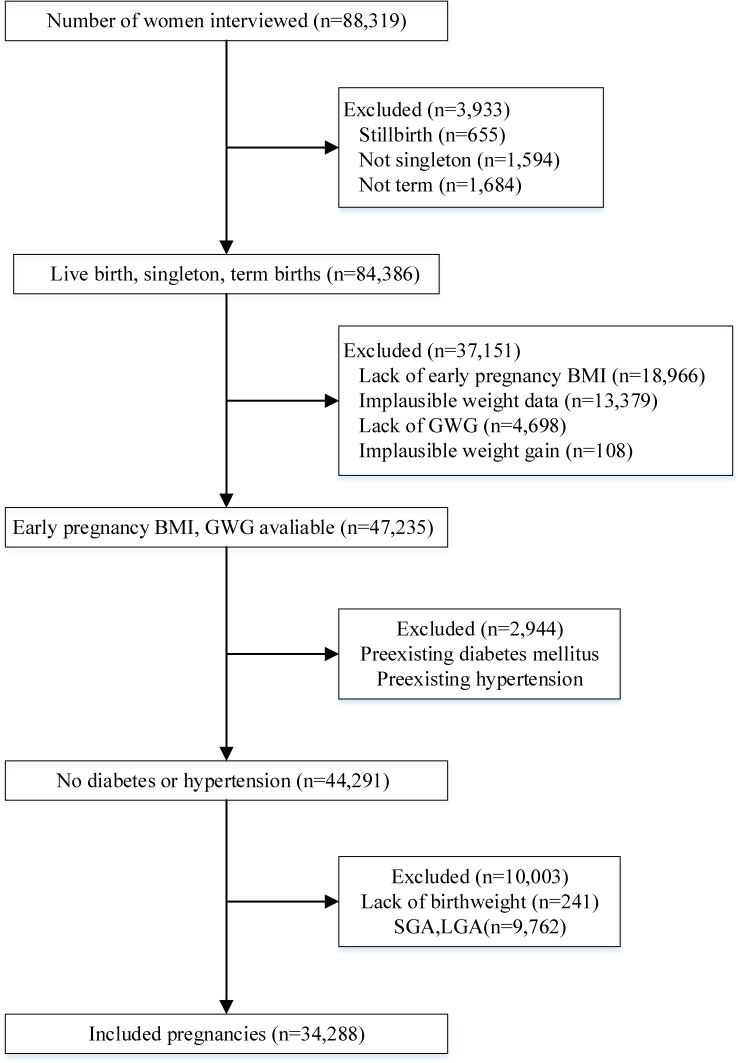
Flowchart for selecting women for study of gestational weight gain of China, 2013–2015.

A total of 88,319 pregnant women were recruited between October 2013 and September 2015 in the MNHMS. We excluded women with multiple births, preterm births, or stillbirths; those whose early pregnancy BMI and plausible weight gain data were missing; and those with pre-existing hypertension or type 1 or 2 diabetes mellitus. This left 44,399 women in the sample. We then excluded women who had newborns without records of birth weight or with abnormal birth weight (either small or large for GA). Finally, we excluded women with implausible weight observations. We therefore included 34,288 women and 253,026 weight observations in the analysis (exclusions are detailed in Figure 1).

Of the participants, 23,091 (67.3%) had a normal BMI during early pregnancy, 6,486 (18.9%) were underweight, 3,817 (11.1%) were overweight, and 894 (2.6%) were obese. Corresponding numbers of weight measurements were 48,397, 167,924, 29,502, and 7,203, respectively. Overall, the median number of weight measurements was 8 (interquartile range, 5–11) per pregnant woman, and the mean GA at first prenatal visit was 9.5 (SD, 2.39) weeks. The characteristics of the 34,288 women and infants who participated in the study are detailed in Table 1.

**Table 1.** Characteristics of 34,288 women and infants, 2013–2015

		Value
Women		
	Age, years, mean (SD)	26.8 (4.3)
	Height, cm, mean (SD)	158.9 (4.8)
	Weight, kg, mean (SD)	53.0 (8.0)
	Early pregnancy BMI, kg/m^2^, mean (SD)	21.0 (2.9)
	Underweight (%)	6,486 (18.9)
	Normal weight (%)	23,091 (67.3)
	Overweight (%)	3,817 (11.1)
	Obese (%)	894 (2.6)
	Education ≥15 years (%)	16,687 (48.7)
	Han ethnicity (%)	32,111 (93.7)
	Cesarean section (%)	13,121 (38.3)
	Nulliparous (%)	21,579 (62.9)
	Gestational age at first visit, weeks, mean (SD)	9.50 (2.39)
	Gestational age at delivery, weeks, mean (SD)	39.25 (1.14)
	Weight measurements (IQR)	8 (5–11)
	Underweight (IQR)	8 (5–11)
	Normal weight (IQR)	8 (5–11)
	Overweight (IQR)	9 (6–11)
	Obese (IQR)	9 (7–11)
Infants		
	Sex, male (%)	17,488 (51.0)
	Birthweight, g, mean (SD)	3,225.2 (272.2)
	Length, cm, mean (SD)	49.9 (1.0)

BMI, body mass index; IQR, interquartile range; SD, standard deviation.

Equations for the smoothed mean values and SDs of weight gain by GA in each BMI category (on the log scale) are shown in Table 2. The constant added to all weight measurements was 3. According to the Akaike information criterion, the knot numbers for best models based on restricted cubic splines were all 8 in underweight, normal weight, overweight, and obese women. The locations of knots are provided in eTable 1.

**Table 2.** Equations for the smoothed mean and standard deviation of weight gain for gestational age in each BMI category

BMI category		Regression equation
Underweight	Mean	1.136975+0.0060688^*^GAs1+0.512251^*^GAs2-1.145976^*^GAs3+0.7481886^*^GAs4-0.0717789^*^GAs5+0.1023281^*^GAs6-0.7902897 ^*^GAs7
	SD	Sqrt(0.2213189+0.000171^*^(GA)^2+2^*^(0.0054634)^*^GA+0.0121334)

Normal weight	Mean	1.141124+0.0094918^*^GAs1+1.071354^*^GAs2-1.690335^*^GAs3+0.7131089^*^GAs4-0.0621935^*^GAs5+0.1044355^*^GAs6-0.6980647^*^GAs7
	SD	Sqrt(0.2214575+0.0001768^*^(GA)^2+2^*^(-0.0054049)^*^GA+0.0136551)

Overweight	Mean	1.302762-0.0003153^*^GAs1+3.656517^*^GAs2-4.300733^*^GAs3+0.6899292^*^GAs4+0.0400375^*^GAs5-0.0641639 ^*^GAs6-0.1666943 ^*^GAs7
	SD	Sqrt(0.2519704+0.0002095^*^(GA)^2+2^*^(-0.0060106)^*^GA+0.0164559)

Obese	Mean	1.504155-0.0194209^*^GAs1+1.798848^*^GAs2-2.404819^*^GAs3+0.6415784^*^GAs4-0.0174574^*^GAs5+0.1425709^*^GAs6-0.5427454^*^GAs7
	SD	Sqrt(0.2431786+0.0002477^*^(GA)^2+2^*^(-0.0061593)^*^GA+0.0228958)

BMI, body mass index; GA, gestational age; SD, standard deviation; Sqrt, square root.

^*^Equations are used to estimate weight gain on the log scale (natural log).

^**^GAs1, GAs2, and so on are derived dependent variables by applying restricted cubic spline to obtain more fitted smooth curves. The number of derived dependent variables (eg, GAs1, GAs2) depends on the optimal number of knots.

**Figure 2.**
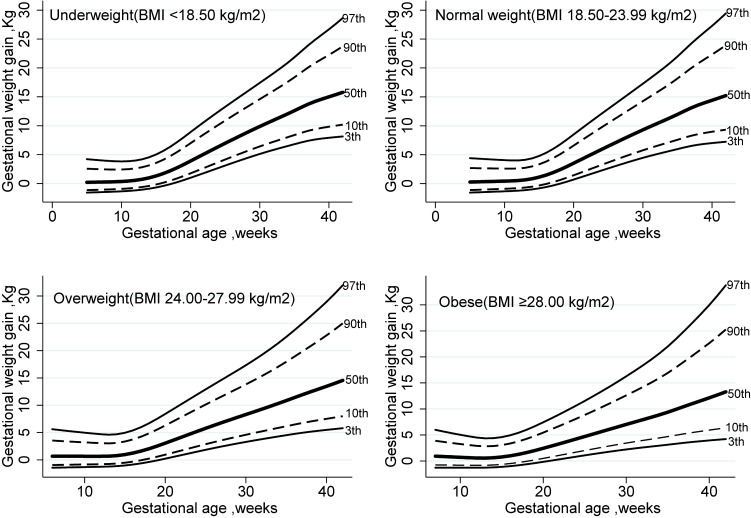
Smoothed percentiles of gestational weight gain at 3^rd^, 10^th^, 50^th^, 90^th^, and 97^th^ percentiles estimated from multilevel linear regression in 34,288 Chinese women within four BMI categories. BMI, body mass index.

Table 3 shows mean values, SDs (log scale), and selected percentiles (3rd, 10th, 50th, 90th, and 97th) of the smoothed week-specific GWG for normal weight women (BMI 18.50–23.99) by GA. The results for underweight, overweight, and obese women are presented in eTable 2, eTable 3, and eTable 4, respectively. The graphical representations of tables for underweight, normal weight, overweight, and obese women are shown in Figure 2. The median values for GWG were 15.0 kg, 14.4 kg, 13.5 kg, and 12.1 kg in underweight, normal weight, overweight, and obese women at 40 weeks, respectively; this median decreased as BMI increased.

**Table 3.** Smoothed mean, standard deviation, and selected percentiles of gestational weight gain for normal weight women (BMI 18.50–23.99 kg/m^2^) according to gestational age

			Percentiles for GWG, kg				
	
GA, weeks	Log mean	Log SD	3rd	10th	50th	90th	97th
5	1.188583	0.430678	−1.54559	−1.1086	0.282427	2.696494	4.40803
6	1.198075	0.42026	−1.50252	−1.06493	0.313731	2.674644	4.332867
7	1.207567	0.410009	−1.45866	−1.02068	0.345334	2.654083	4.260751
8	1.217058	0.399937	−1.41406	−0.97587	0.377239	2.634889	4.191781
9	1.22655	0.390058	−1.36876	−0.93056	0.409447	2.61715	4.126069
10	1.236042	0.380387	−1.32283	−0.88481	0.441963	2.60096	4.063737
11	1.246724	0.370942	−1.27428	−0.83608	0.478928	2.593076	4.013267
12	1.264549	0.361738	−1.21242	−0.77106	0.541494	2.626981	4.01628
13	1.296658	0.352795	−1.12262	−0.67184	0.657055	2.744459	4.123796
14	1.348316	0.344134	−0.99047	−0.52108	0.850936	2.982315	4.379673
15	1.417275	0.335776	−0.81272	−0.31553	1.125862	3.341202	4.78261
16	1.499408	0.327745	−0.58917	−0.05563	1.479035	3.813602	5.321522
17	1.590588	0.320064	−0.3204	0.257323	1.906633	4.391054	5.984579
18	1.686689	0.312761	−0.00911	0.619569	2.401567	5.060882	6.755265
19	1.783585	0.305861	0.33845	1.023218	2.951151	5.802953	7.608575
20	1.877148	0.299394	0.710972	1.454541	3.53484	6.586653	8.507535
21	1.964045	0.293386	1.09409	1.896449	4.128101	7.376872	9.410528
22	2.044111	0.287868	1.481865	2.342208	4.722287	8.162746	10.30556
23	2.117973	0.282868	1.871256	2.788663	5.314264	8.941789	11.19079
24	2.186259	0.278414	2.25961	3.233196	5.901847	9.713041	12.0663
25	2.249596	0.274532	2.644782	3.673843	6.483907	10.47717	12.93409
26	2.308613	0.271246	3.025238	4.109396	7.060457	11.23648	13.79814
27	2.363866	0.26858	3.399693	4.538956	7.631976	11.99397	14.66318
28	2.415639	0.266551	3.765646	4.960197	8.196922	12.74974	15.53054
29	2.464144	0.265174	4.120408	5.370563	8.753414	13.5034	16.40096
30	2.509595	0.264459	4.461579	5.76781	9.299944	14.25501	17.27568
31	2.552202	0.264412	4.787066	6.150013	9.835342	15.005	18.15636
32	2.592298	0.265032	5.096132	6.516777	10.36044	15.75648	19.04772
33	2.630672	0.266316	5.392461	6.872833	10.88309	16.52229	19.96588
34	2.668237	0.268254	5.68187	7.22537	11.41453	17.31993	20.93249
35	2.7059	0.270831	5.971282	7.582859	11.96778	18.16955	21.97239
36	2.743795	0.27403	6.261608	7.946679	12.54587	19.07738	23.09418
37	2.778953	0.277829	6.524386	8.283386	13.10215	19.97886	24.22269
38	2.808611	0.282205	6.730301	8.558136	13.58687	20.80351	25.275
39	2.83395	0.287129	6.887544	8.780252	14.01253	21.56875	26.27179
40	2.857127	0.292576	7.01575	8.972713	14.41144	22.32075	27.26813
41	2.879946	0.298516	7.132532	9.156278	14.81331	23.10288	28.31635
42	2.902761	0.30492	7.241644	9.335279	15.22439	23.92509	29.42922

BMI, body mass index; GA, gestational age; GWG, gestational weight gain; SD, standard deviation.

The result of sensitivity analyses showed that 17.2% of women in the normal weight group (3,963/23,091) were excluded and added to the underweight group to form a reclassified group of underweight women (*n* = 10,448, 77,311 weight measurements); 82.8% of the women (19,128/23,091) remained in the normal weight group. In the overweight group, 32.0% of the women (1,220/3,817) were added to the normal weight group to form a reclassified normal weight group (*n* = 20,349, 148,353 weight measurements). Then, 32.0% of overweight women (1,220/3,817) were excluded from the overweight group and 29.2% of women from the obese group (261/894) were added to the overweight group to form a reclassified overweight group (*n* = 2,858, 22,225 weight measurements). Finally, 29.2% of women in the obese group (261/894) were excluded to form a reclassified obese group (*n* = 633, 5,137 weight measurements). The percentile values on the GWG Z-score charts were indistinguishable after we reclassified these groups.

### Assessing internal and external validation

**Figure 3.**
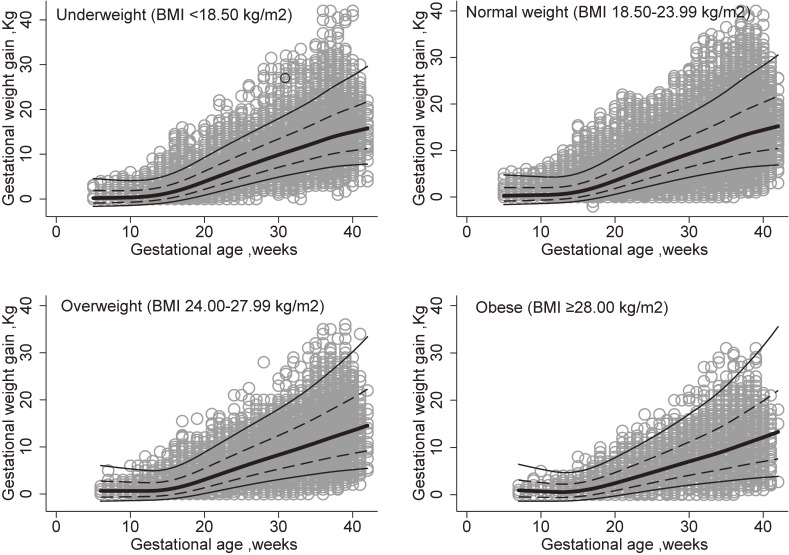
Fitted means and SDs of gestational weight gain for women estimated from multilevel linear regression overlaid on observed measurements in 34,288 Chinese women within four BMI categories. Dotted lines and lines denote the fitted mean ± 1 SD and 2 SD. BMI, body mass index; SD, standard deviation.

The result of internal validation showed that in underweight, normal weight, overweight, and obese women, 72.5% (35,110/48,397), 72.3% (121,419/167,924), 71.3% (21,029/29,502), and 70.8% (5,102/7,203) of crude weight measurements fell within the expected 1 SD (68% expected) of the mean, respectively, and 95.8% (46,370/48,397), 95.9% (160,967/167,924), 96.0% (28,320/29,502), and 95.6% (6,888/7,203) of weight measurements fell within the expected 2 SDs (95% expected) of the mean, respectively. The estimated lines for means, 1 SD, and 2 SDs and crude weight measurements are shown in Figure 3.

The population used to assess the external validity (validation sample set) included 20,458 pregnant women with 146,994 weight observations. Baseline demographic characteristics are shown in eTable 5 and exclusions are detailed in eFigure 1. Z-score distributions of GWG are shown in eFigure 2, which were calculated from multilevel linear models in 20,458 pregnant women with four BMI categories and superimposed on the non-skewed standard normal curve. The result of comparing the fit of the models that were developed using the training set to the crude data of the validation set showed that in underweight, normal weight, overweight, and obese women, 73.3% (18,796/25,648), 70.6% (67,224/95,185), 65.3% (13,300/20,377), and 62.2% (3,598/5,784) of crude weight measurements fell within the expected 1 SD (68% expected) of the mean, respectively, and 95.8% (24,576/25,648), 93.1% (88,625/95,185), 89.2% (18,169/20,377), and 87.7% (5,073/5,784) of weight measurements fell within the expected 2 SDs (95% expected) of the mean, respectively. The 5th, 10th, 15th, 25th, 75th, 85th, 90th, and 95th percentiles were selected to calculate sensitivity, specificity, and the Youden index for each BMI category; the 10th and 90th percentiles are shown in Table 4 and the other percentiles are shown in eTable 6. The specificities of measurements at the 10th percentile for the four BMI categories were 100%, as were the specificities for measurements at the 5th, 15th, and 25th percentiles. The specificities of measurements within the 90th percentile were above 90%, similar to those of measurements in the 75th, 85th, and 95th percentiles. The sensitivities of measurements within the 10th percentile were 94.4%, 72.3%, 52.8%, and 45.9% for the underweight, normal weight, overweight, and obese categories, respectively, which decreased as the BMI increased. The sensitivities of measurements within the 5th, 15th, and 25th percentiles increased as the percentiles increased. The sensitivities of measurements within the 90th percentile for all four BMI categories were 100%, the same as those of measurements within the 75th, 85th, and 95th percentiles.

**Table 4.** Sensitivity, specificity, and Youden index by four BMI categories for selected percentiles

Percentile	BMI category	EZ^a^	OZ^b^	TP	FP	FN	TN	SE (%)	SP (%)	YI
10^th^	Underweight	−1.28	−1.32	2,583	0	154	22,964	94.4	100.0	0.94
	Normal weight	−1.28	−1.54	9,251	0	3,547	82,293	72.3	100.0	0.72
	Overweight	−1.28	−1.99	2,043	0	1,823	16,616	52.8	100.0	0.53
	Obese	−1.28	−2.12	581	0	686	4,570	45.9	100.0	0.46
90^th^	Underweight	1.28	1.01	1,372	1,207	0	23,122	100.0	95.0	0.95
	Normal weight	1.28	1.02	5,439	4,059	0	85,593	100.0	95.5	0.96
	Overweight	1.28	0.98	1,065	940	0	18,392	100.0	95.1	0.95
	Obese	1.28	1.02	335	229	0	5,241	100.0	95.8	0.96

BMI, body mass index; EZ, expected Z-score; FN, false negative; FP, false positive; OZ, observed Z-score; SE, sensitivity; SP, specificity; TN, true negative; TP, true positive; YI, Youden index.

^a^Expected Z-score was estimated by the model.

^b^Observed Z-score was actual Z-score of the validation set.

## DISCUSSION

In our study, we developed percentiles and Z-score charts that described GWG by GA of healthy Chinese women with BMI categories based on Chinese population norms. These models for weight gain by GA performed well for Chinese women in internal and external validation analyses, which showed that the sensitivities, specificities, and Youden indexes of the Z-scores were acceptable. Our Z-score charts could, therefore, be used for epidemiologic studies within the Chinese population to estimate the unbiased association between GWG and maternal and offspring outcomes. However, because optimal Z-scores and percentile ranges remain to be established with more sufficient clinical evidence, these charts should be used only to encourage clinicians to closely monitor a woman with a very low or very high Z-score, rather than recommending immediate behavioral alterations.

In our study, median weight gains at 40 weeks were within the ranges of the IOM recommendations for underweight and normal weight categories and were higher than the IOM recommendations for overweight and obese categories, which was consistent with other studies.^15^^,^^21^ In other words, the IOM recommended ranges fell within the 25th and 75th percentiles of our Z-score charts for underweight and normal weight women and fell below the 50th percentile for overweight and obese women. The common reference that is currently used in China to recommend GWG in clinical practice and epidemiological studies is the IOM recommendations. However, total GWG recommendations proposed by the IOM are linked to pregnancy duration, and the application of a single standard during the whole pregnancy would introduce bias for weight management. Therefore, compared with IOM recommendations, our Z-score charts may be most suited for guidance in monitoring GWG in pregnant women in China.

Previous studies have provided few relative GWG Z-score charts.^12^^–^^16^ A study based at the Magee-Womens Hospital in Pittsburgh reported mean singleton GWG at 40 weeks of 16.4 kg for normal weight women^12^ and 15.8 kg for overweight women^14^; these figures are higher than those observed in our study (14.4 kg for normal weight women and 13.5 kg for overweight women). A cohort study, the INTERGROWTH-21st Project, reported a standard GWG at 40 weeks of 13.7 kg in normal weight women who came from eight countries, including China^13^; this is about 0.7 kg less than the average GWG seen in our study. The INTERGROWTH study did not develop Z-score charts for women who were underweight, overweight, and obese. In addition, in this cohort study, 5 GA windows were selected for 8 countries to obtain 40 comparisons to assess variations in GWG across countries. Only the data from China showed high (>0.5) standardized site differences (see Figure 3 of the original paper^13^), which indicated that Chinese women might be different from other study populations; this can probably be attributed not only to socioeconomic and cultural factors, but also to actual biological differences. Thus, it remains debatable whether this international standard is suitable for Chinese women. Most previous studies used populations with a small sample size (<5,000), were not population-based, and did not obtain Z-score charts for women in different BMI categories. A population-based cohort study performed in Sweden obtained GWG values at 40 weeks that differed from ours by 0.2 to 1.4 kg for different BMI categories.^15^ However, it is difficult to compare our charts with previous charts because study designs, methods, and populations are different.

To date, no GWG Z-score charts have been externally validated, although it is essential for practical use to validate and calibrate new models. Whether the models can be applied to populations other than the one studied depends on their performance outside the training sample, that is, external validation.^22^ Temporal validation is one category of external validation,^23^ which is better than internal validation.^22^ In our study, we assessed the temporal validation of our GWG Z-score charts. After we developed the GWG models with a first sample of pregnant women, we assessed the models’ external validation among pregnant women who delivered in different years. Because no optimal cut-offs of GWG Z-score charts were available, we selected several percentiles to assess external validation by means of calculating the sensitivity, specificity, and Youden index. The analysis of external validation showed that our GWG Z-score charts had high sensitivities and specificities for Chinese underweight, normal weight, and overweight women.

In this population-based study, we developed Chinese GWG Z-score charts, according to WHO recommendations,^19^ with a large sample size of healthy pregnant women who had good maternal and perinatal outcomes. Compared with total GWG recommendations, Z-score charts could provide means and SDs throughout most of the pregnancy for clinicians or pregnant women to monitor the progress in GWG. Although a single low or high Z-score might be uninformative, more concern could be taken when low or high Z-scores occur repeatedly or if a very low or very high Z-score appears, so that clinicians could give their patients advice or treatment.

One limitation of our study is that the early pregnancy BMI, rather than pre-pregnancy BMI, was used to classify women. This introduces the possibility of misclassification bias. However, it is not feasible to measure the pre-pregnancy weight of women in large population studies, especially in low-risk women, and self-reported pre-pregnancy weight is generally unreliable due to its ability to introduce error or recall bias. The results of the sensitivity analyses indicated that the possibility of misclassification was small and acceptable. Early pregnancy BMI was used as the baseline, in fact, pregnant women have 0–2 kg weight gain in the first trimester,^8^ which also caused flat weight gain curve lines and the less reliable results in the early period (<13 weeks). However, the weight of the first prenatal care in early pregnancy is used by obstetric clinicians as individual-specific baseline to monitor GWG in second and third trimester, which may mean that our study has more practical application value.

Another limitation is that the sample size of obese pregnant women in our study was comparatively small, because the rate of obesity is low in Chinese pregnant women (the rate of obesity in our study was 2.6% [*n* = 894]). The small sample size did not allow us to classify obese women into different classes and resulted in lower specificities for validity of Z-scores of obese women within the 5th and 10th percentiles in comparison with other BMI categories. Further study is needed in a larger sample of obese pregnant women to ensure the robustness of GWG model. In addition, the failure to identify and remove pregnant women who have some pregnancy complications that are reportedly associated with excessive or insufficient GWG is one of the limitations of our study. We do not have sufficient evidence to exclude pregnant women, because the diagnostic evidence associated with these complications was not collected, such as medication records and surgery records. Therefore, further comprehensive study design and information collection needs to be done.

In conclusion, we have described Z-score charts for GWG in a population-based follow-up study of Chinese women with healthy pregnancy outcomes, which provides a tool for epidemiological studies to explore the association between GWG and adverse pregnancy outcomes. Moreover, these Z-score charts provide greater sensitivity for monitoring a woman’s GWG than measurements such as total GWG or the rate of GWG. The Z-score charts were developed using Chinese women with healthy pregnancy outcomes; however, they are not necessarily equal to the optimal GWG. Further epidemiological studies are needed to make the associations of Z-scores and long-term outcomes, such as maternal weight retention and childhood and adolescent obesity, and to establish thresholds for optimal weight gain.

## APPENDIX A. SUPPLEMENTARY DATA

eAppendix 1. Data collection details

eFigure 1. Flowchart for selecting validation women included in study of gestational weight gain of China, 2015–2016. BMI, body mass index; GWG, gestational weight gain; LGA, large for gestational age; SGA, small for gestational age.

eFigure 2. Z-score distributions of GWG calculated from multilevel linear models in 20,458 Chinese women in 2015–2016 with four BMI categories, which superimposed on the non-skewed standard normal curve. Lines denote the observed normal distribution curves and dotted lines denote the standard normal distribution curves.

eTable 1. Knot number and knot location of restricted cubic splines models in four BMI categories

eTable 2. Smoothed mean, standard deviation, and selected centiles of gestational weight gain for underweight women (BMI <18.50 kg/m^2^) according to gestational age

eTable 3. Smoothed mean, standard deviation, and selected centiles of gestational weight gain for overweight women (BMI 24.00–27.99 kg/m^2^) according to gestational age

eTable 4. Smoothed mean, standard deviation, and selected centiles of gestational weight gain for obese women (BMI >28.00 kg/m^2^) according to gestational age

eTable 5. Characteristics of 20,458 validation women and infants, 2015–2016

eTable 6. Results of the sensitivity, specificity and Youden index for four BMI categories for selected centiles
