# Supplementary material for: Gestational Weight Gain Charts by Gestational Age and Body Mass Index for Chinese Women: A Population-Based Follow-up Study
Source: J Epidemiol. 2020 Aug 5;30(8):345–53. doi: 10.2188/jea.JE20180238 (PMC7348073; doi:10.2188/jea.JE20180238)

eAppendix 1. Data collection details

The unified perinatal health care cards were given for pregnant women at the first prenatal care visit and withdrawn at the 28th-day postpartum examination between all study districts or counties. All maternal information about before pregnancy, all prenatal care visits and delivery and neonatal information were included in perinatal health care cards. Maternal information was measured and recorded by well-trained local medical institution staff who participated in this project. The staff of the district and county maternal and child health institutions was responsible for input related data into MNHMS monthly in the form of electronic records. In order to ensure the quality of data, the rate of missing report and intact rate of information were checked routinely. The district and county maternal and child health institutions conduct self-examination monthly, the municipal maternal and child health institutions conduct checking quarterly, and the provincial maternal and child health institutions conduct checking annually. The content of maternal information included: maternal social demography characteristics, such as region, age, education, and nation; last menstrual period (LMP), pregnancy history, and medical history. The content of prenatal care visits included: height, weight, blood pressure measurements; required biochemical examinations, such as blood routine, hepatic and renal function examination, as well as B-ultrasound and Down’s syndrome screening. The content of delivery information included: delivery mode and obstetric complications of pregnant women and birth weight, birth length, and Apgar score of neonates.

eTable 1. Knot number and knot location of restricted cubic splines models in four BMI categories

BMI categories	Knot number	Knot location
Underweight	8	7,13,20,26,31,35,37,40
Normal weight	8	7, 13,20,26,31,35,37,40
Overweight	8	12, 13,20,26,31,35,37,40
Obese	8	11, 13,20,26,31,35,37,40

BMI, body mass index.

eTable 2. Smoothed mean, standard deviation, and selected centiles of gestational weight gain for underweight women (BMI <18.50 kg/m^2) according to gestational age

Gestational age, weeks	logmean	logSD	Centiles for GWG, kg				
			3th	10th	50th	90th	97th
5	1.167319	0.427894	-1.56868	-1.14179	0.213366	2.556806	4.214109
6	1.173388	0.41719	-1.53054	-1.10469	0.232927	2.514555	4.112664
7	1.179457	0.406625	-1.49177	-1.06719	0.252606	2.473599	4.014486
8	1.185996	0.396209	-1.4517	-1.0284	0.273946	2.436546	3.922878
9	1.195357	0.385956	-1.40655	-0.98356	0.304738	2.416128	3.853876
10	1.210363	0.375878	-1.35136	-0.9265	0.354704	2.427547	3.826232
11	1.233837	0.36599	-1.28036	-0.85021	0.43438	2.486571	3.858969
12	1.268599	0.356307	-1.18665	-0.74641	0.555867	2.61068	3.972816
13	1.317473	0.346847	-1.06147	-0.6047	0.733973	2.820797	4.192321
14	1.382228	0.337629	-0.89543	-0.41413	0.98377	3.137351	4.540938
15	1.460426	0.328672	-0.6854	-0.17156	1.307796	3.560892	5.017418
16	1.548575	0.319999	-0.43033	0.123568	1.704761	4.086376	5.613869
17	1.643183	0.311634	-0.13034	0.470475	2.171603	4.706577	6.320076
18	1.740758	0.303602	0.212184	0.86572	2.701664	5.409551	7.12052
19	1.837809	0.295929	0.59126	1.301741	3.282759	6.176066	7.991425
20	1.930845	0.288646	0.996043	1.765385	3.895333	6.977287	8.898175
21	2.01706	0.281781	1.412732	2.240309	4.516195	7.780508	9.802316
22	2.096399	0.275367	1.835363	2.719776	5.136815	8.575235	10.69241
23	2.169492	0.269434	2.260681	3.20041	5.753832	9.35879	11.56647
24	2.236969	0.264017	2.685826	3.679392	6.364903	10.13015	12.42457
25	2.299462	0.259147	3.108451	4.154583	6.968814	10.89001	13.26881
26	2.3576	0.254856	3.52684	4.624639	7.565568	11.64085	14.10341
27	2.41195	0.251173	3.939527	5.088545	8.155692	12.38589	14.93342
28	2.462811	0.248125	4.343793	5.543849	8.737762	13.12565	15.76075
29	2.51042	0.245736	4.736728	5.987886	9.310095	13.8603	16.58689
30	2.55501	0.244025	5.115714	6.418328	9.871427	14.59056	17.41393
31	2.596818	0.243007	5.478507	6.833243	10.42096	15.31769	18.24458
32	2.636172	0.242691	5.824103	7.232083	10.95966	16.04522	19.08409
33	2.673779	0.243078	6.15556	7.618937	11.49464	16.78489	19.9472
34	2.710435	0.244166	6.477885	8.000079	12.03582	17.5522	20.85298
35	2.746941	0.245946	6.797269	8.383109	12.59486	18.36495	21.82319
36	2.78337	0.248402	7.113695	8.768372	13.17343	19.22737	22.86394
37	2.816891	0.251515	7.39711	9.121152	13.72477	20.07685	23.90343
38	2.844903	0.255261	7.61704	9.405862	14.19988	20.84646	24.86426
39	2.868605	0.259611	7.782665	9.632877	14.61244	21.55482	25.76821
40	2.890154	0.264538	7.915426	9.826908	14.99607	22.24838	26.66982
41	2.911344	0.270008	8.034513	10.01019	15.38148	22.97032	27.62018
42	2.932535	0.27599	8.144135	10.18747	15.77517	23.73044	28.63161

GWG, gestational weight gain; SD, standard deviation.

eTable 3. Smoothed mean, standard deviation, and selected centiles of gestational weight gain for overweight women (BMI 24.00–27.99 kg/m^2) according to gestational age

Gestational age, weeks	logmean	logSD	Centiles for GWG, kg				
			3th	10th	50th	90th	97th
7	1.300555	0.441071	-1.40491	-0.91246	0.671333	3.456746	5.450102
8	1.30024	0.430888	-1.37443	-0.88574	0.670176	3.371128	5.286423
9	1.299924	0.420957	-1.34415	-0.85937	0.669019	3.28867	5.129776
10	1.299609	0.411296	-1.31417	-0.83342	0.667862	3.209421	4.980158
11	1.299294	0.401924	-1.28458	-0.80796	0.666706	3.133441	4.837574
12	1.298978	0.392861	-1.2555	-0.78309	0.66555	3.060793	4.702046
13	1.303327	0.38413	-1.21874	-0.7484	0.681525	3.019559	4.609014
14	1.330174	0.375755	-1.14108	-0.66221	0.7817	3.117414	4.693299
15	1.380073	0.367758	-1.01621	-0.51731	0.975194	3.364931	4.965627
16	1.448096	0.360165	-0.84588	-0.3166	1.255007	3.747068	5.40485
17	1.529312	0.353003	-0.63179	-0.06277	1.615002	4.251125	5.99338
18	1.618791	0.346299	-0.37708	0.239855	2.046983	4.862093	6.711348
19	1.711602	0.340078	-0.08796	0.583364	2.537824	5.5583	7.531271
20	1.802815	0.334369	0.224759	0.954377	3.0667	6.307368	8.413207
21	1.88838	0.329197	0.547339	1.336241	3.608654	7.071926	9.311849
22	1.967767	0.324589	0.874025	1.722286	4.154681	7.839972	10.21351
23	2.041326	0.320569	1.201539	2.10897	4.70081	8.607522	11.11446
24	2.109405	0.317159	1.526621	2.492823	5.243337	9.371162	12.01177
25	2.172358	0.314378	1.846139	2.870578	5.778956	10.12819	12.9034
26	2.23053	0.312245	2.157161	3.239224	6.304794	10.8766	13.78815
27	2.284324	0.310772	2.457355	3.596475	6.819046	11.61594	14.66674
28	2.334347	0.309969	2.746011	3.941979	7.322717	12.34987	15.54478
29	2.381252	0.30984	3.023414	4.276549	7.818442	13.08437	16.43063
30	2.425698	0.310387	3.290661	4.601933	8.310124	13.82716	17.33473
31	2.468341	0.311607	3.549604	4.920739	8.802848	14.58765	18.26956
32	2.509749	0.31349	3.802249	5.235731	9.301844	15.37546	19.24784
33	2.550181	0.316026	4.04905	5.547754	9.809427	16.19585	20.2771
34	2.589793	0.319198	4.290037	5.857101	10.32701	17.05276	21.3633
35	2.628753	0.322989	4.525554	6.164412	10.85649	17.95085	22.51336
36	2.667012	0.327376	4.754481	6.468498	11.39688	18.89051	23.72909
37	2.703684	0.332336	4.969076	6.760012	11.93465	19.85282	24.98867
38	2.737966	0.337844	5.161599	7.029442	12.45551	20.81716	26.26789
39	2.770457	0.343874	5.335605	7.281014	12.96593	21.79433	27.58097
40	2.802043	0.350397	5.49767	7.522695	13.47828	22.80457	28.95389
41	2.83347	0.357389	5.65386	7.761904	14.00436	23.86777	30.41264
42	2.864899	0.36482	5.805605	8.000369	14.54728	24.99061	31.96716

GWG, gestational weight gain; SD, standard deviation.

eTable 4. Smoothed mean, standard deviation, and selected centiles of gestational weight gain for obese women (BMI >28.00 kg/m^2) according to gestational age

Gestational age, weeks	logmean	logSD	Centiles for GWG, kg				
			3th	10th	50th	90th	97th
8	1.348788	0.428227	-1.28496	-0.773	0.852752	3.665323	5.654997
9	1.329367	0.418653	-1.28723	-0.7889	0.778651	3.457507	5.33632
10	1.309946	0.409461	-1.29073	-0.80576	0.705973	3.259224	5.035164
11	1.290525	0.400676	-1.29554	-0.82363	0.634695	3.070195	4.750856
12	1.273243	0.392326	-1.2981	-0.83793	0.57242	2.902766	4.498778
13	1.268795	0.38444	-1.28021	-0.82569	0.556564	2.817541	4.355041
14	1.287154	0.377045	-1.22369	-0.76434	0.622464	2.869517	4.387357
15	1.326858	0.370173	-1.12758	-0.65324	0.769181	3.053756	4.587365
16	1.383582	0.363852	-0.99448	-0.49609	0.989166	3.355447	4.934829
17	1.453004	0.358111	-0.82686	-0.2963	1.275941	3.762457	5.41347
18	1.5308	0.352979	-0.62815	-0.0583	1.621875	4.2617	6.006364
19	1.612648	0.348483	-0.40389	0.211022	2.016077	4.835831	6.691816
20	1.694224	0.344648	-0.16274	0.501089	2.442419	5.460205	7.439626
21	1.771967	0.341496	0.084963	0.799434	2.882412	6.10735	8.216594
22	1.845369	0.339046	0.335332	1.101654	3.330434	6.770301	9.015112
23	1.914683	0.337313	0.586448	1.405806	3.784791	7.448347	9.835369
24	1.980165	0.336308	0.836432	1.710013	4.243939	8.141085	10.67799
25	2.042068	0.336039	1.083501	2.012517	4.706526	8.848448	11.54403
26	2.100645	0.336506	1.326027	2.311732	5.171439	9.570744	12.43505
27	2.156131	0.337706	1.562478	2.606164	5.637652	10.30839	13.35274
28	2.208675	0.339633	1.791144	2.894061	6.103647	11.061	14.29783
29	2.258407	0.342273	2.01038	3.173699	6.567839	11.82799	15.27078
30	2.305455	0.34561	2.21872	3.443516	7.028745	12.60882	16.2721
31	2.349953	0.349625	2.414943	3.702184	7.485079	13.40314	17.3025
32	2.392196	0.354294	2.59896	3.949711	7.937486	14.21346	18.36622
33	2.433159	0.359591	2.774963	4.191376	8.394826	15.05525	19.48362
34	2.473995	0.36549	2.948974	4.434771	8.869778	15.95036	20.68335
35	2.515848	0.371962	3.12782	4.68858	9.377096	16.92468	21.99951
36	2.559218	0.378978	3.315143	4.957593	9.925705	17.99552	23.45607
37	2.602022	0.386507	3.498194	5.225947	10.49099	19.12594	25.00883
38	2.642178	0.394521	3.662761	5.475599	11.04375	20.26997	26.60139
39	2.68014	0.40299	3.810661	5.708617	11.58713	21.43378	28.24284
40	2.717008	0.411886	3.948618	5.933369	12.13497	22.64177	29.96589
41	2.753695	0.421183	4.082724	6.157566	12.70054	23.9184	31.80399
42	2.790378	0.430855	4.214279	6.382852	13.28718	25.27201	33.77043

GWG, gestational weight gain; SD, standard deviation.

eTable 5. Characteristics of 20,458 validation women and infants, 2015–2016

		Value
Women		
	Age, years, mean (SD)	27.3 (4.4)
	Height, cm, mean (SD)	159.0 (4.8)
	Weight, kg, mean (SD)	54.2 (8.7)
	Early pregnancy BMI, kg/m^2, mean (SD)	21.4 (3.2)
	Underweight (%)	3,403 (16.6)
	Normal weight (%)	13,333 (65.2)
	Overweight (%)	2,909 (14.2)
	Obese (%)	813 (4.0)
	Education ≥15 years (%)	6,727 (32.9)
	Han ethnicity (%)	18,667 (91.2)
	Caesarean section (%)	6,889 (33.7)
	Nulliparous (%)	10,627 (51.9)
	Gestational age at first visit, weeks, mean (SD)	11.7 (4.7)
	Gestational age at delivery, weeks, mean (SD)	39.4 (1.1)
	Weight measurements (IQR)	7 (5–9)
	Underweight (IQR)	8 (5–10)
,	Normal weight (IQR)	7 (5–9)
	Overweight (IQR)	7 (5–9)
	Obese (IQR)	7 (5–10)
infants		
	Sex, male, (%)	10,532 (51.5)
	Birthweight, g, mean (SD)	3,241.1 (261.9)
	Length, cm, mean (SD)	50.0 (0.9)

BMI, body mass index; IQR, interquartile range; SD, standard deviation.

eTable 6. Results of the sensitivity, specificity and Youden index for four BMI categories for selected centiles

Centiles	BMI category	EZ	OZ	TP	FP	FN	TN	SE (%)	SP (%)	YI
5th	Under weight	-1.645	-1.75	1236	0	309	24156	80.0	100.0	0.80
	Normal weight	-1.645	-2.13	4768	0	3432	86891	58.1	100.0	0.58
	Over weight	-1.645	-2.67	1037	0	1773	17672	36.9	100.0	0.37
	Obese	-1.645	-2.68	288	0	631	4918	31.3	100.0	0.31
15th	Under weight	-1.04	-1.08	3857	0	157	21687	96.1	100.0	0.96
	Normal weight	-1.04	-1.21	14285	0	3152	77654	81.9	100.0	0.82
	Over weight	-1.04	-1.55	3023	0	1929	15445	61.0	100.0	0.61
	Obese	-1.04	-1.73	867	0	714	4224	54.8	100.0	0.55
25th	Under weight	-0.67	-0.71	6285	0	245	19171	96.2	100.0	0.96
	Normal weight	-0.67	-0.77	23999	0	2013	69079	92.3	100.0	0.92
	Over weight	-0.67	-1.01	5007	0	1979	13411	71.7	100.0	0.72
	Obese	-0.67	-1.13	1415	0	710	3680	66.6	100.0	0.67
75th	Under weight	0.67	0.49	4834	1565	0	19302	100.0	92.5	0.93
	Normal weight	0.67	0.50	17983	5719	0	71389	100.0	92.6	0.93
	Over weight	0.67	0.43	3542	1517	0	15338	100.0	91.0	0.91
	Obese	0.67	0.41	1038	415	0	4352	100.0	91.3	0.91
85th	Under weight	1.04	0.82	2386	1204	0	22111	100.0	94.8	0.95
	Normal weight	1.04	0.78	9242	5998	0	79851	100.0	93.0	0.93
	Over weight	1.04	0.74	1800	1362	0	17235	100.0	92.7	0.93
	Obese	1.04	0.78	549	329	0	4927	100.0	93.7	0.94
95th	Under weight	1.645	1.32	588	700	0	24413	100.0	97.2	0.97
	Normal weight	1.645	1.34	2230	2494	0	90367	100.0	97.3	0.97
	Over weight	1.645	1.30	429	591	0	19377	100.0	97.0	0.97
	Obese	1.645	1.38	156	115	0	5534	100.0	98.0	0.98

BMI, body mass index; EZ, expected Z-score; FN, false negative; FP, false positive; OZ, observed Z-score; SE, sensitivity; SP, specificity; TN, true negative; TP, true positive; YI, Youden index.

e**Figure 1.** Flowchart for selecting validation women included in study of gestational weight gain of China, 2015–2016. BMI, body mass index; GWG, gestational weight gain; LGA, large for gestational age; SGA, small for gestational age.

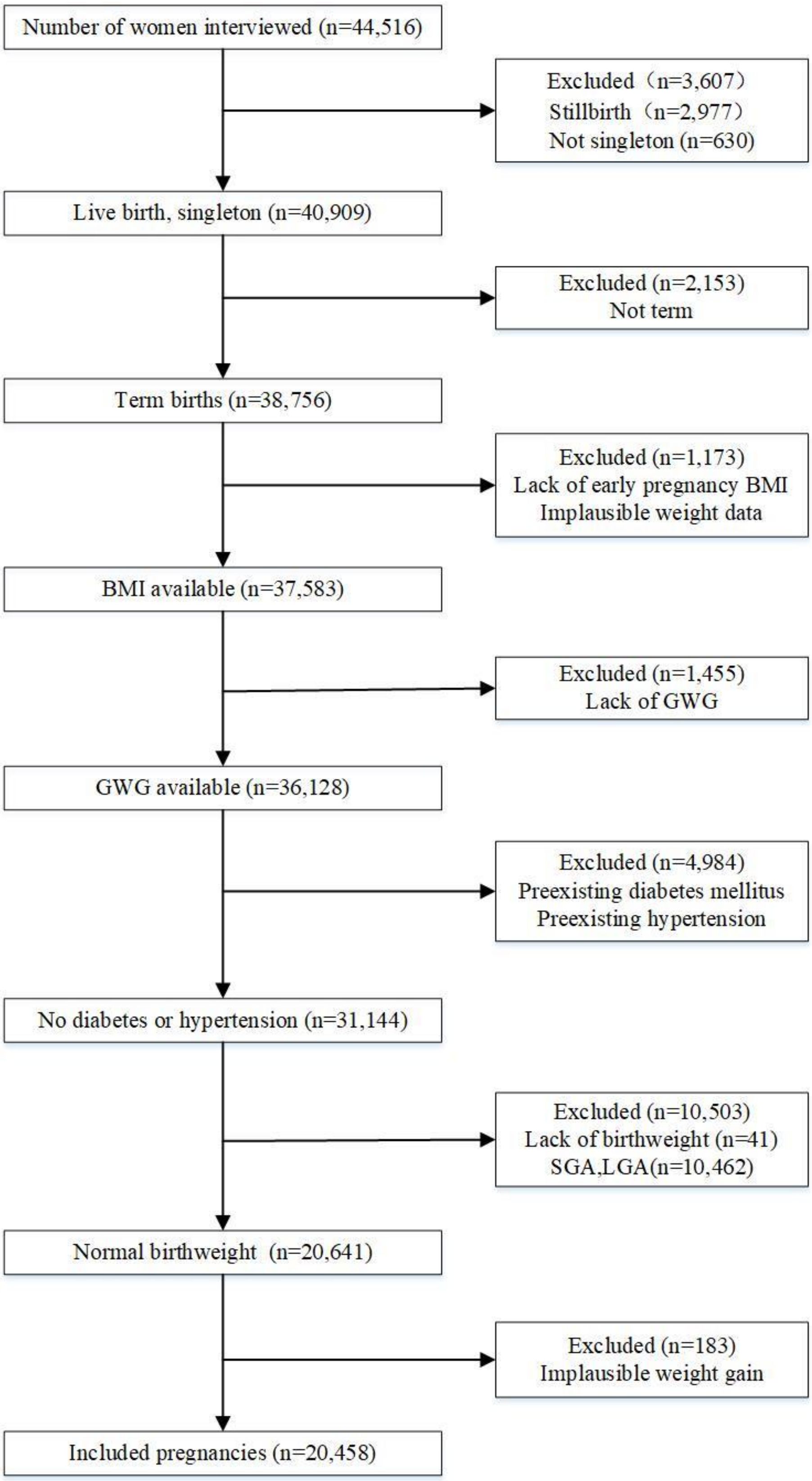

eFigure 2. Z-score distributions of GWG calculated from multilevel linear models in 20,458 Chinese women in 2015–2016 with four BMI categories, which superimposed on the non-skewed standard normal curve. Lines denote the observed normal distribution curves and dotted lines denote the standard normal distribution curves.

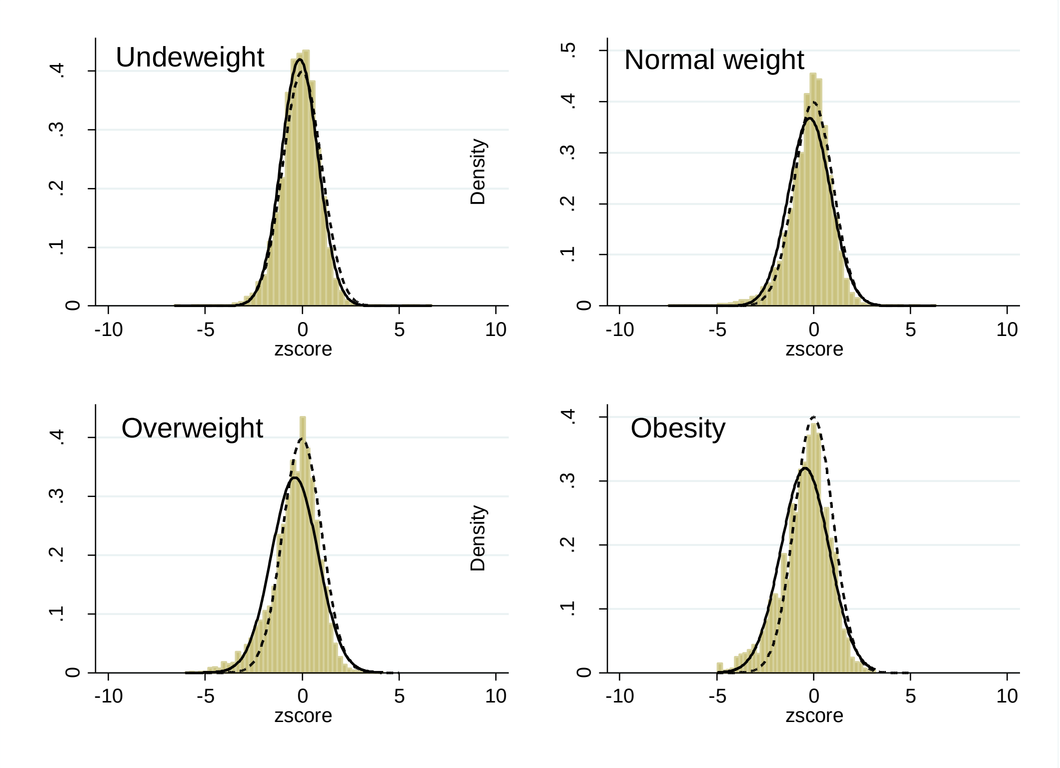

Supplement: Supplementary file 1 [file je-30-345-s001.pdf]
